# Relationship between Oxygen Uptake, Heart Rate, and Perceived Effort in an Aquatic Incremental Test in Older Women

**DOI:** 10.3390/ijerph17228324

**Published:** 2020-11-11

**Authors:** Luana Siqueira Andrade, Ana Carolina Kanitz, Mariana Silva Häfele, Gustavo Zaccaria Schaun, Stephanie Santana Pinto, Cristine Lima Alberton

**Affiliations:** 1 Neuromuscular Assessment Laboratory, Physical Education School, Federal University of Pelotas, Pelotas 96055-630, Brazil; andradelu94@gmail.com (L.S.A.); marianaesef@hotmail.com (M.S.H.); gustavoschaun@hotmail.com (G.Z.S.); tetisantana@yahoo.com.br (S.S.P.); 2 Physical Education, Physiotherapy and Dance, Federal University of Rio Grande do Sul, Porto Alegre 90690-200, Brazil; ana_kanitz@yahoo.com.br; 3 UAB Center for Exercise Medicine, University of Alabama at Birmingham, Birmingham, AL 35205, USA

**Keywords:** water-based exercises, water aerobics, cardiorespiratory, rate of perceived exertion, aging, maximum test

## Abstract

Different parameters can be used to control the intensity of aerobic exercises, a choice that should consider the population and exercise environment targeted. Therefore, our study aimed to verify the relationship between oxygen uptake (VO_2_), heart rate (HR), rating of perceived exertion (RPE), and cadence during an aquatic incremental test in older women. Nine older women (64.3 ± 4.4 years) engaged in a water-based aerobic training performed an aquatic incremental test using the stationary running exercise (cadence increases of 15 b·min^−1^ every 2 min) until participants’ volitional exhaustion. VO_2_, HR, and RPE data were measured, and the percentage of peak VO_2_ (%VO_2peak_) and percentage of maximal HR (%HR_max_) were calculated. Linear and polynomial regression analyses were performed (α = 0.05). Polynomial regressions revealed the best adjustments for all analyses. Data showed a significant relationship (*p* < 0.001) between %VO_2peak_ and %HR_max_ (*r* = 0.921), %VO_2peak_ and RPE (*r* = 0.870), and %HR_max_ and RPE (*r* = 0.878). Likewise, significant relationships between cadence (*p* < 0.001) and %VO_2peak_ (*r* = 0.873), %HR_max_ (*r* = 0.874), and RPE (*r* = 0.910) were also observed. In summary, the physiological, subjective, and mechanical variables investigated were highly associated during an aquatic incremental test to exhaustion in older women. Therefore, these different parameters can be employed to adequately prescribe water-based programs according to preference and availability.

## 1. Introduction

Advancing age is associated with a decline in cardiorespiratory fitness [1,2,3], and regular aerobic exercise practice is a cornerstone intervention to mitigate this effect in older individuals [4,5]. To maximize potential benefits arising from aerobic training, correct exercise intensity prescription is a crucial factor. This can be achieved by using both objective (i.e., physiological or mechanical) or subjective (i.e., rating of perceived exertion—RPE) parameters of intensity control [6,7,8]; however, their choice should take into consideration the exercise modality and the population at hand.

The aquatic environment favors the practice of physical exercises, since water resistance is multidirectional and promotes an overload against movements performed in all directions [9]. Additional physical properties of water, such as hydrostatic pressure and water’s thermal conditions also promote important physiological effects in the human body [10,11], leading to benefits in hemodynamic, neuroendocrine, and metabolic parameters. Moreover, buoyancy promotes biomechanical alterations, such as a low ground reaction force during water-based exercises performance [12]. Collectively, these characteristics favor adherence of older individuals to this type of exercise, whose environment appears to be safe for those individuals who usually present cardiovascular and musculoskeletal disorders, highly prevalent in the elderly [13,14]. In fact, the effectiveness of exercise programs in the aquatic environment, prescribed to older individuals on the basis of heart rate (HR) and RPE, has already been demonstrated in several investigations analyzing parameters of physical fitness and quality of life [15,16,17,18,19,20].

Although physiological parameters, such as HR and oxygen uptake (VO_2_), are typically recognized as the most reliable objective parameters to control exercise intensity, their use in the aquatic environment requires attention. Since the maximal HR and VO_2_ are affected by water immersion [21,22], a maximal incremental test with water-based exercises is necessary in order to adequately determine the maximal and submaximal values for an adequate prescription. An alternative is to use mechanical parameters such as cadence (CAD), which can also be employed to control exercise intensity during water aerobics. Following this prescription, songs that have the intended cadence(s) are chosen, and time is synchronized with the execution of a segmental action of a water-based exercise [23]. Although exercising at a certain cadence allows one to modulate exercise load, it does not take inter-individual differences into account, since a given music cadence may represent different percentages of maximal effort for different individuals [24]. Subjective parameters like RPE, on the other hand, are a simple and low-cost tool that is also typically used for exercise prescription in the water environment [19,25]. This is particularly true for special populations like older individuals, who are frequently on medications (e.g., beta blockers) that can influence cardiovascular responses to exercise [26]. However, extra time and effort are necessary to provide enough information before the beginning of water-based training programs, so that the method may be considered valid and suitable for reaching the target intensity.

Actually, HR and RPE are the most frequently used parameters for exercise training prescription in water-based aerobic programs because both allow the training load to be individualized and are easy to implement in water aerobics classes composed of groups of older women [17,18,19,25]. In this regard, previous studies have observed significant linear relationships between the aforementioned parameters, such as between VO_2_ and HR [27,28], VO_2_ and RPE [27,29], and HR and RPE [29,30], in young adult women performing aquatic exercise by different modalities at submaximal intensities. More recently, however, evidence has suggested that the relationships between some of these parameters are not always linear and may be polynomial [24,31,32]. Specifically, polynomial relationships were observed between VO_2_ and water-walking treadmill load [31], percentage of peak VO_2_ (%VO_2peak_) and RPE [24], as well as between %VO_2peak_, percentage of maximal HR (%HR_max_), RPE and CAD [32] with different aquatic incremental protocols until exhaustion in young adults.

To the best of our knowledge, no investigation to date has analyzed the associations between physiological, mechanical, and subjective parameters of intensity control in water-based exercises in older women. Understanding the relationship between these parameters can assist exercise practitioners to more adequately prescribe water-based exercise intensity for older individuals, which is the population that most often seeks this exercise modality. Thus, the purpose of the present study was to verify the relationship between VO_2_, HR, RPE, and CAD during an aquatic incremental test in older women using the stationary running exercise and also to verify the best adjustment for these relationships (i.e., linear vs. polynomial). A secondary aim was to define %HR_max_, RPE, and CAD values for different %VO_2peak_, corresponding to different training zones. Based on the available evidence, our initial hypotheses were that high and significant polynomial relationships would be observed between the physiological, mechanical, and subjective parameters.

## 2. Materials and Methods

### 2.1. Participants

Nine older women (from 60 to 75 years old) who participated in the Effects of Two Water-Based Aerobic Training Programs in Elderly Women Study (WATER Study; NCT03289091) volunteered and were included in the present investigation after completing the water-based aerobic training program. In the WATER Study, participants completed either a continuous or an interval water-based intervention for three months [19,20], with two 45 min weekly sessions, and those individuals who had a history of cardiovascular disease (except for controlled hypertension) and/or osteoarticular injuries, which prevented them from being able to exercise, were not included. In addition, participants who were taking beta blockers or who were smokers were also not included in the present investigation. The present study was approved by the Federal University of Pelotas Local Ethics Research Committee (CAAE: 69931817.5.0000.5313), and all participants were informed of all the details and procedures of the study prior to signing a written informed consent form.

### 2.2. Procedures

Participants attended two separate sessions. During their first visit, anthropometric measures were taken, and the experimental procedures were explained in detail to the participants in the laboratory. Body weight and height were measured using a digital scale and a stadiometer (WELMY, Santa Bárbara d’Oeste, Brasil). Based on these values, the body mass index (BMI) was calculated using the formula body mass (kg)/stature^2^ (m). In their second visit, participants performed a water-based maximal incremental test at the pool of a sports club affiliated to the Federal University of Pelotas, using the stationary running exercise, and the outcomes of interest were assessed. Stationary running is typically employed to investigate the behavior of several physiological outcomes in the aquatic environment [21,22,24,32,33,34], since it is one of the most common exercises performed in water aerobics sessions and is relatively easy to teach and perform. Starting from the upright position, the exercise has two phases. The first one corresponds to the unilateral hip and knee flexion up to 90°, and the second one to the unilateral hip and knee extension. The movement of the lower limbs is performed alternately (i.e., when one limb is flexed, the contralateral limb is simultaneously extended), with each phase corresponding to a beat of the metronome used to control cadence. Upper limb movement is performed to provide adequate balance during exercise performance by alternating shoulder semi-flexion and extension, while the elbows are flexed at 90°. Prior to the test, participants were familiarized during 3 min with the testing procedures, exercise technique, and cadence control within the rhythm imposed by the metronome, which served as a warm-up before the test performance. Participants also received standardized instructions on the Borg RPE 6–20 Scale [35]. All the aforementioned procedures were performed in order to standardize the data collection for all participants, since the exercise used in the protocol, as well as the RPE Scale had been previously employed in the WATER trial [19,20]. In addition, participants were asked to refrain from caffeine, alcohol, or any other stimulant and any type of physical exercise 24 h before the experimental protocol.

All tests were carried out in a pool where participants were immersed to the level of the xiphoid process, and water temperature was kept at ~32 °C. Tests were monitored by three instructors, one inside and two outside the pool, with one of the two responsible for controlling exercise technique and range of motion, as well as for providing constant feedback throughout the test. Specifically, the maximal incremental test began at 70 b⋅min^−1^, and cadence was increased by 15 b⋅min^−1^ every 2 min up to participants’ volitional exhaustion or when they were not able to maintain adequate cadence or range of motion. Cadences were reproduced and controlled by a digital app (Metronome).

During the incremental test, gas exchange data were obtained and averaged every three breaths using a mixing-chamber gas analyzer (VO_2_000, MedGraphics^®^, Ann Arbor, MI, USA), which was calibrated prior to each session according to the manufacturer’s specifications. HR was measured telemetrically (FT1, Polar, Kempele, Finland) every 30 s, and Borg’s RPE at every minute. The scale (21 × 29.7 cm) was presented to the participants so that they could choose the number corresponding to their RPE. Tests were validated when at least two of the following criteria were achieved: VO_2_ plateau despite an increase in exercise intensity; respiratory exchange ratio (RER) greater than 1.15; respiratory rate greater than 35 breaths per min [36]; or RPE greater or equal to 18.

Throughout the incremental test, the average of the last three VO_2_ measures at each stage was considered as representative of that stage, whereas stage-specific HR and RPE corresponded to the last value at the end of each stage. At the last stage, VO_2_ values were time-averaged every 15 s, and the highest 15 s interval obtained was considered as VO_2peak_, whereas HR_max_ was determined as the highest HR value observed during the test. Only stages that lasted more than 50% of the stage duration were included. Mean HR and VO_2_ for each stage were also determined and expressed as %VO_2peak_ and %HR_max_ based on maximal values individually obtained in the aquatic incremental test. Finally, %VO_2peak_ responses were used to determine five training zones, namely, 50–59%, 60–69%, 70–79%, 80–89%, and 90–99% VO_2peak_, and their corresponding mean values of %HR_max_, RPE, and CAD were determined.

### 2.3. Statistical Analysis

Descriptive data are presented as mean ± standard deviation (SD). Data normal distribution was verified using the Shapiro–Wilk test, and the relationships between VO_2_, %VO_2peak_, HR, %HR_max_, RPE, and CAD were tested using linear and polynomial regression analyses. The test presenting the best fit for each analysis (i.e., linear or polynomial) was adopted, and r values were classified according to the recommendations from Safrit and Wood [37], i.e., 0–0.19 as no correlation, 0.20–0.39 as low correlation, 0.4–0.59 as moderate correlation, 0.6–0.79 as moderately high correlation, and 0.8–1.0 as high correlation. All analyses were processed in the SPSS software (version 20.0, IBM Corporation, Armonk, NY, USA) adopting an alpha level equal to 5%.

## 3. Results

All participants completed the sessions, and no adverse events or safety concerns were observed throughout the tests. Mean age, body mass, height, and BMI corresponded to 64.3 ± 4.4 years, 69.7 ± 7.7 kg, 151.1 ± 4.6 cm, 30.6 ± 4.1 kg⋅m^−2^.

**Figure 1:**
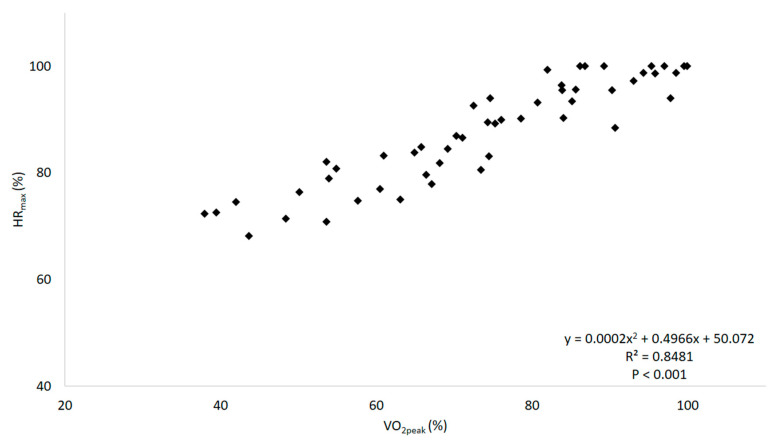
Relationship between percentage of peak oxygen uptake (%VO_2peak_) and percentage of the maximal heart rate (%HR_max_) during a progressive test with the stationary running exercise in aquatic environment.

**Figure 2:**
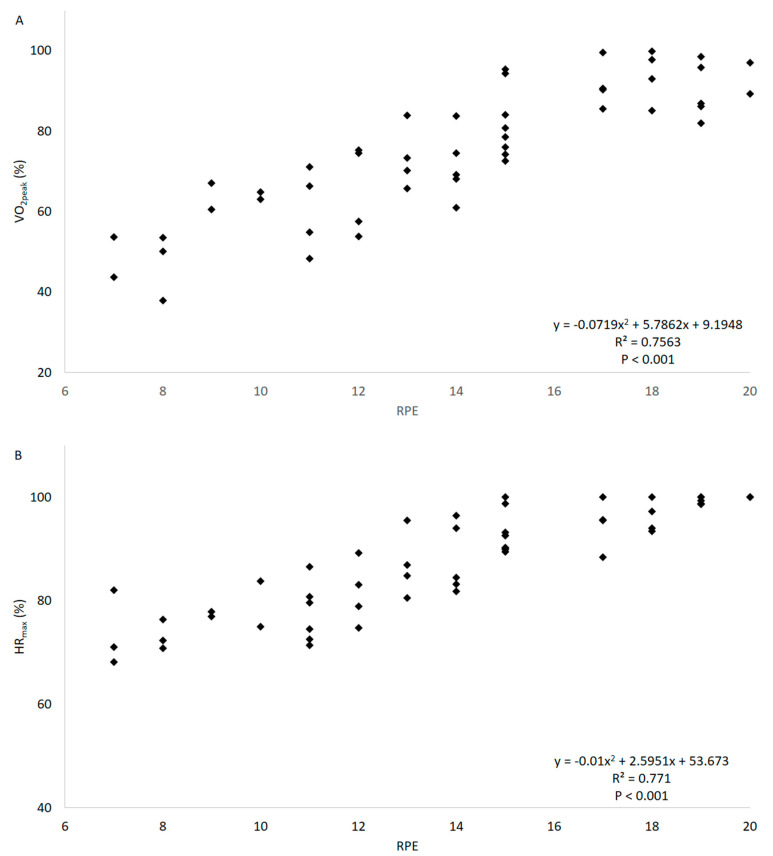
Relationship between RPE and the physiological variables %VO_2peak_ (**A**) and %HR_max_ (**B**) during a progressive test with the stationary running exercise in aquatic environment.

**Figure 3:**
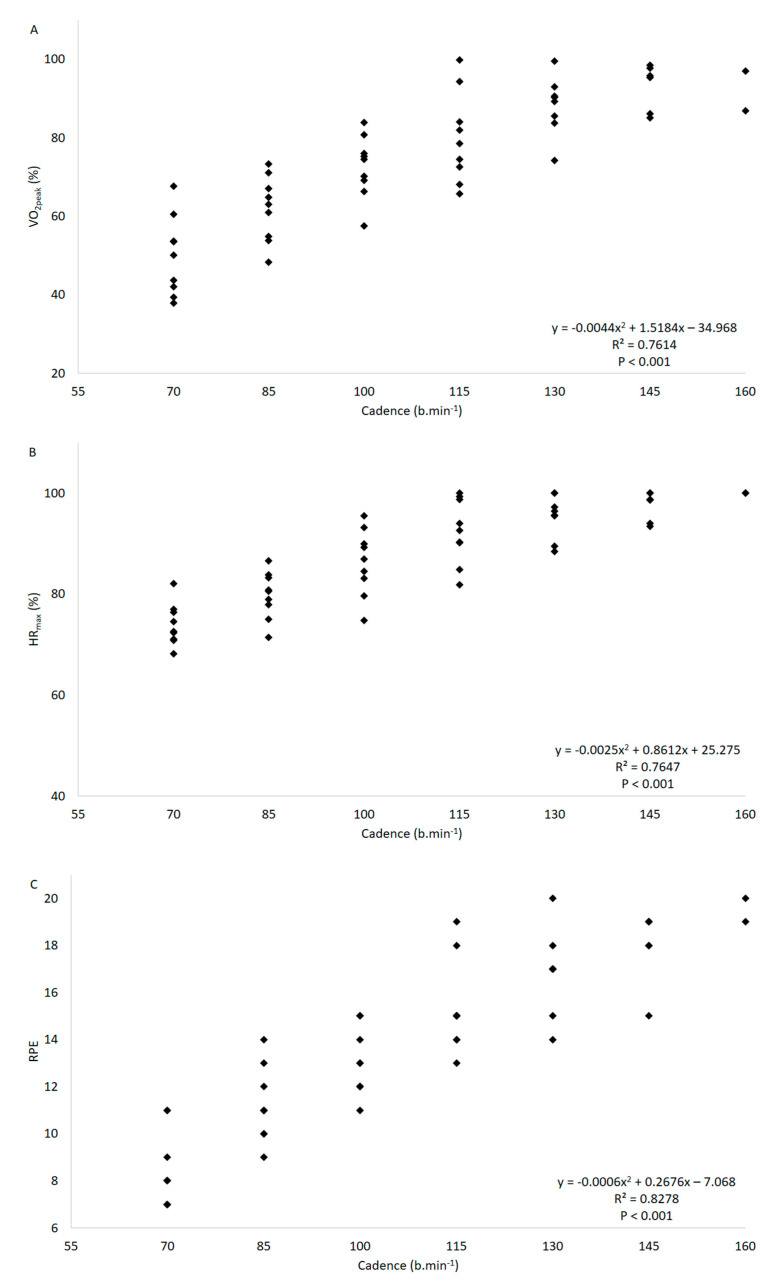
Relationship between cadence and %VO_2peak_ (**A**), %HR_max_ (**B**), and RPE (**C**) during a progressive test with the stationary running exercise in aquatic environment.

Polynomial regression models showed the best adjustment in all analysis, as presented in Table 1. Significant relationships (*p* < 0.001) were observed between the physiological variables %VO_2peak_ and %HR_max_ (Table 1; Figure 1), as well as VO_2_ and HR (Table 1). Significant relationships (*p* < 0.001) were also observed between the subjective and the physiological variables, such as between RPE and %VO_2peak_ and (Table 1; Figure 2A), VO_2_ (Table 1), %HR_max_ (Table 1; Figure 2B), and HR (Table 1). Moreover, the relationships between CAD and the physiological and subjective variables were also significantly associated (*p* < 0.001), such as between CAD and %VO_2peak_ (Table 1; Figure 3A), VO_2_ (Table 1), %HR_max_ (Table 1; Figure 3B); HR (Table 1), and RPE (Table 1; Figure 3C). Lastly, the mean %HR_max_, RPE, and cadence descriptive responses for the training zones that were determined according to the %VO_2peak_ results are shown in Table 2.

## 4. Discussion

The main findings of the present study were the high and significant polynomial correlations observed between physiological (VO_2_, HR), subjective (RPE), and mechanical (CAD) parameters, during an aquatic incremental maximal test using the exercise of stationary running performed by older women. In addition, %HR_max_, RPE, and CAD reference values were also identified for different training zones based on the %VO_2peak_, which may be a valuable tool for the prescription of water-based exercise sessions in older women, who are the main practitioners of this modality.

In the present study, polynomial regressions showed the best adjustments in all analyses; however, it should be stressed that the observed differences in the resulting *r* values were small in comparison to those observed for the linear correlation for all outcomes (see Table 1). Therefore, the authors reinforce the significant relationships observed between physiological, subjective, and mechanical parameters, regardless of the type of adjustment. Although VO_2_ is considered the gold standard for controlling exercise intensity during aerobic training programs, its use outside the laboratory is limited. Therefore, alternative parameters like HR and RPE are widely used to prescribe exercise intensity. This is especially true in the aquatic environment, because both these parameters can be easily applied to individualized exercise intensity, even when training sessions are performed in groups [17,18,19,25,38].

Regarding the relationship between physiological outcomes, a high polynomial correlation was observed between %VO_2peak_ and %HR_max_ (*r* = 0.921) during the water-based incremental test performed by the older women. Such findings are in agreement to those reported by David et al. [32], who also found a high polynomial correlation (*r* = 0.858) between the aforementioned outcomes using a similar protocol in young women. In addition, a moderately high polynomial correlation was observed between VO_2_ and HR (*r* = 0.739) in the present study. Previous studies in which exercises were performed only at submaximal intensities reported linear relationship between VO_2_ and HR (values were not relativized by the maximum) during water-walking on a flowmill (*r* = 0.999) [39,40] and a nonmotorized aquatic treadmill (*r* = 0.710) [28], as well as during water-based exercises that included stationary running (*r* = 0.782–0.857) [27,41].

The current results support the higher *r* values observed for physiological parameters when expressed relative to maximal values (i.e., %VO_2peak_ and %HR_max_) rather than to absolute values (i.e., VO_2_ and HR) (see Table 1). In addition, it should be highlighted that the maximal values should be obtained in the specific environment considered (i.e., aquatic incremental test), since the use of maximum values derived from a dry-land incremental protocol may impact on the data used in this analysis, as demonstrated by Alberton et al. [22]. Therefore, we postulate that regardless of the type of relationship (linear vs. polynomial), protocol (submaximal vs. maximal), aquatic modality (water walking vs. water-based exercises), there is a moderately high to high correlation between the physiological variables investigated herein, supporting the use of HR to prescribe exercise intensity during water-based exercise sessions.

When the physiological outcomes were correlated to a subjective parameter, high polynomial correlations were found between RPE and %VO_2peak_ or %HR_max_ (*r* = 0.870–0.878), and moderately high polynomial correlations between RPE and VO_2_ or HR (*r* = 0.713–0.756). Our findings are in agreement with previous studies that also observed significant polynomial relationships between physiological variables and RPE in aquatic incremental tests to exhaustion in young women, observing *r* = 0.858–0.871 between %VO_2peak_ and RPE [24,32] and *r* = 0.823 between %HR_max_ and RPE [32]. Regarding submaximal protocols, moderate to moderately high linear correlations were observed between VO_2_ and RPE (*r* = 0.436–0.600) and between HR and RPE (*r* = 0.550–0.650) during water-based exercises [27,29,30] or water-walking [28]. In these latter studies, absolute physiological parameters were used, resulting in lower *r* values. Therefore, the present findings support the use of RPE for prescribing water-based aerobic exercise intensities, since this subjective parameter highly reflects the physiological intensity.

Additionally, cadence imposed by the musical rhythm of a song may also be used as a way to control exercise intensity during water-based exercise sessions [23,42]. The findings of the present study demonstrated high polynomial correlations between CAD and the physiological variables investigated (*r* = 0.673–0.874) and also between CAD and RPE (*r* = 0.910), demonstrating that the load provided by the drag force in aquatic environment is quadratically increased in accordance to the general fluids equation. Our findings corroborate those of David et al. [32], who also observed high polynomial associations (*r* = 0.848–0.878), as well as of Barbosa et al. [42], who verified moderately high to high linear relationships (*r* = 0.610–0.850), both studies being conducted in young women. Although significant relationships between CAD and physiological and subjective parameters were demonstrated, it should be highlighted that CAD does not take into consideration biological individuality, and, therefore, a pre-determined cadence may represent different training zones for different individuals [24].

The better adjustments observed in the polynomial regression models may be attributed to the components which influence drag force, as presented in the general fluids equation: Fd = 0.5 × ρ × A × V^2^ × Cd, where Fd = drag force, ρ = density, A = projected area, V = velocity, and Cd = drag force coefficient [43]. Based on this equation, the drag force provided by a fluid (e.g., water) to motion is strongly influenced by the velocity of motion, which is squared and directly proportional to it, reflecting on the increase observed in the physiological and subjective parameters during an incremental protocol. Therefore, this should be taken into consideration for the prescription of exercise intensity in the aquatic environment and also for determining the progression of training load in water-based training programs, especially those involving higher intensities.

The American College of Sports Medicine presents guidelines for developing and maintaining the cardiorespiratory capacity in apparently healthy adults [44]. Regarding endurance conditioning, values of 57–63% of HR_max_ and 9–11 of RPE are indicated for intensities corresponding to 37–45% of VO_2max_ (light), values of 64–76% of HR_max_ and 12–13 of RPE for intensities corresponding to 46–63% of VO_2max_ (moderate), values of 77–95% of HR_max_ and 14–17 of RPE for intensities corresponding to 64–90% of VO_2max_ (vigorous), and values ≥96% of HR_max_ and ≥18 of RPE for intensities greater than 90% of VO_2max_ (near-maximal to maximal). Nevertheless, these recommendations are based on data from exercises performed on dry land, and their usage for water-based exercises is limited. In addition, the guidelines for exercise in older adults do not even show a relationship between different intensity parameters [4]. On the other hand, %VO_2peak_ and RPE recommendations in the aquatic environment are available only for young women [24]. Therefore, in order to help aquatic training instructors to more easily control and prescribe the intended intensity in water-based training programs, we provided %HR_max_, RPE, and CAD references corresponding to different training zones based on the %VO_2peak_ (see Table 2). These values may prove valuable and helpful for practitioners to more adequately prescribe water-based exercise programs for older women in real-world settings, such as clubs and recreational centers.

A limitation of the present study is that, although the stationary running exercise is widely used in water aerobics, it was the only exercise investigated. A water-based program including other exercises that involve different muscle groups and types of displacement may influence the investigated outcomes and, therefore, the association between them. In addition, our sample included older women who were trained in water aerobics for three months and who were well-familiarized with the exercises and the subjective parameters; therefore, such findings may not be completely extrapolated to other populations, such as untrained individuals or men, for example. Additionally, the sample size may also be mentioned as a possible limitation. Accordingly, we suggest that future studies investigate these parameters in larger and/or different populations, also using different exercises to confirm and expand our findings. In addition, the inclusion of parameters derived from HR reserve, such as the percentages of HR reserve, in future analyses could provide valuable information for the prescription of water-based exercises.

## 5. Conclusions

The present study showed that the physiological (VO_2_, HR), subjective (RPE), and mechanical (CAD) parameters investigated were highly associated during an aquatic incremental test performed in older women using the stationary running exercise. Based on these findings, it seems that different parameters can be employed to adequately prescribe water-based training sessions according to preference and availability. In addition, %HR_max_, RPE, and CAD reference values corresponding to different training zones based on the %VO_2peak_ were also identified and provided, which may be a valuable and useful tool for water-based exercise intensity prescription in older women in real-world settings.

## Figures and Tables

**Table 1:** Relationships between oxygen uptake (VO_2_), heart rate (HR), rating of perceived exertion (RPE), and cadence (CAD) using linear and polynomial regression analyses.

Relationships	Linear		Polynomial	
	*r*	*p*	*r*	*p*
%VO_2peak_ × %HR_max_	0.921	<0.001	0.921	<0.001
VO_2_ × HR	0.734	<0.001	0.739	<0.001
%VO_2peak_ × RPE	0.868	<0.001	0.870	<0.001
VO_2_ × RPE	0.711	<0.001	0.713	<0.001
%HR_max_ × RPE	0.878	<0.001	0.878	<0.001
HR × RPE	0.755	<0.001	0.756	<0.001
%VO_2peak_ x CAD	0.855	<0.001	0.873	<0.001
VO_2_ × CAD	0.663	<0.001	0.673	<0.001
%HR_max_ × CAD	0.858	<0.001	0.874	<0.001
HR × CAD	0.789	<0.001	0.795	<0.001
RPE × CAD	0.902	<0.001	0.910	<0.001

%VO_2peak_—percentage of peak oxygen uptake; %HR_max_—percentage of the maximal heart rate.

**Table 2:** Descriptive statistics (mean ± SD) of the %HR_max_, RPE, and CAD corresponding to different training zones during the stationary running exercise.

Intensity (%VO_2peak_)	%HR_max_	RPE	CAD
	Mean ± SD	Mean ± SD	Mean ± SD
50–59%	77.3 ± 4.2	9.7 ± 2.3	80.0 ± 12.2
60–69%	79.9 ± 4.6	11.1 ± 2.5	91.0 ± 16.1
70–79%	88.2 ± 4.1	13.5 ± 1.5	104.5 ± 14.2
80–89%	96.4 ± 3.4	16.9 ± 2.5	127.0 ± 19.7
90–99%	97.4 ± 3.6	17.5 ± 1.6	135.5 ± 13.9
